# Automatic Extraction of Structural and Non-Structural Road Edges from Mobile Laser Scanning Data

**DOI:** 10.3390/s19225030

**Published:** 2019-11-18

**Authors:** Mengmeng Yang, Xianlin Liu, Kun Jiang, Jingzhong Xu, Peng Sheng, Diange Yang

**Affiliations:** 1 State Key Laboratory of Automotive Safety and Energy, School of Vehicle and Mobility, Tsinghua University, Beijing 100084, China; 2 Chinese Academy of Surveying and Mapping, Beijing 100830, China; 3 School of Remote Sensing and Information Engineering, Wuhan University, Wuhan 430079, China

**Keywords:** remote sensing, mobile laser scanning, road edge detection, topological network

## Abstract

Accurate road information is important for applications involving road maintenance, intelligent transportation, and road network updates. Mobile laser scanning (MLS) can effectively extract road information. However, accurately extracting road edges based on large-scale data for complex road conditions, including both structural and non-structural road types, remains difficult. In this study, a robust method to automatically extract structural and non-structural road edges based on a topological network of laser points between adjacent scan lines and auxiliary surfaces is proposed. The extraction of road and curb points was achieved mainly from the roughness of the extracted surface, without considering traditional thresholds (e.g., height jump, slope, and density). Five large-scale road datasets, containing different types of road curbs and complex road scenes, were used to evaluate the practicality, stability, and validity of the proposed method via qualitative and quantitative analyses. Measured values of the correctness, completeness, and quality of extracted road edges were over 95.5%, 91.7%, and 90.9%, respectively. These results confirm that the proposed method can extract road edges from large-scale MLS datasets without the need for auxiliary information on intensity, image, or geographic data. The proposed method is effective regardless of whether the road width is fixed, the road is regular, and the existence of pedestrians and vehicles. Most importantly, the proposed method provides a valuable solution for road edge extraction that is useful for road authorities when developing intelligent transportation systems, such as those required by self-driving vehicles.

## 1. Introduction

Road edges are an indispensable part of road information [1,2] and play an important role in the construction of surveying and mapping geographic information. Fine, accurate, efficient, and fast automatic road edge extraction technology promotes the development of digital cities, emergency commands, road maintenance, and 3-D maps, and allows for the rapid development of new technologies, such as intelligent navigation, self-driving vehicles, and intelligent transportation. The mobile laser scanning (MLS) system is a multi-platform, multi-mode, multi-sensor integrated technology tool that comprises laser scanners, navigation systems, and high-resolution digital cameras. Navigation systems include an inertial navigation system and a global navigation satellite system [3,4,5,6,7,8,9,10,11,12]. The MLS can rapidly, continuously, and reliably capture high-accuracy 3-D spatially referenced and other information (e.g., intensity, color) from road infrastructure and surrounding road corridor environments [9]. A number of previous studies have focused on extraction and recognition of various objects based on MLS data, such as roads [1,2,9], road markings [13,14,15,16,17], and others [10,11,18,19,20,21]. At present, the technology to extract road edges [22,23], which is key to acquiring road geometry information and road objects [9], cannot simultaneously satisfy both structural (different road curb types) and non-structural road types (such as that of a grass-soil road). Meanwhile, the existing methods rely on traditional parameters to extract road edge information, such as height jump [24], point density [25], and slope [26,27]. Thus, the extraction of accurate, efficient, and complete road edge information, especially for complex structural and non-structural road conditions with severe occlusions, varying curbstones, and large data processing requirements, remains a significant challenge [2,22].

In this study, we propose a robust method to extract structural and non-structural road edges from MLS data with complex road conditions. The proposed method uses a topological network of laser points placed between scan lines and the vertical auxiliary surface to extract accurate road edges. The remainder of this paper is organized as follows: Related literature on road extraction from laser points is discussed in Section 2. The key technology and algorithms are elucidated in Section 3; specifically, the construction of the topological network is presented in Section 3.1, the recognition of ground points is presented in Section 3.2, the detections of curb points are shown in Section 3.3, and the extraction of road edges is shown in Section 3.4. Detailed experimental analysis is discussed in Section 4, with conclusions presented in Section 5. 

## 2. Previous Work

There are primarily two challenges for extracting road edges from MLS data. On the one hand, high-density and high-accuracy point clouds, with many types of objects (e.g., vehicles, buildings, trees, poles, and pedestrians) captured, contain holes and occlusions that increase the difficulty of data extraction. On the other hand, roads in different regions have different road structures, which has a great impact on the performance of the algorithm. Most research has focused on structural road types, while only a few proposed methods are applicable for non-structural road types. Additionally, structural roads have different types of curbstones and non-structural roads do not have curbstones on both sides. Most non-structural roads have two sides that comprise grassland, wheat fields, gravel, or some other non-defined transition. Previous methods have performed road edge extraction based on laser points or intensity images generated from laser points. 

For extraction directly from laser points, many studies have used horizontal plane characteristics based on profile segmentation [28], a quasi-flat zone method with a region adjacency graph representation [29], or an associative Markov network [30] to extract road surface information. These methods are time-consuming since they search for adjacent points from a large number of unorganized laser points. Subsequently, studies have used horizontal lines to extract road surface information. For example, Manandhar and Shibasaki [31] used a height histogram analysis of each scan line. This method; however, is only appropriate for flat roads with little variations in height. McElhinney et al. [32] introduced a road edge segmentation algorithm based on fit lines of road cross-sections by calculating the slope of the spline and finding the change and start points. Miraliakbari, et al. [24] extracted road curbs of a structural road with smooth road surface and road curbstone based on height differences and height histograms. Extraction results of these methods do not have a sufficiently resolution [33]. Moreover, several factors, such as road width, slope, and density [25], were included in the road edge extraction. Abuhadrous et al. [34] subsequently altered this method to consider road width, slope, and curvature to construct the histogram. Yoon et al. [35] used road edge seeds to extract the road surface from surrounding non-road surfaces. Road edges depend on the parameters of slope [26,27], road width [31], intensity, and vehicle proximity [36]. Zhang [37] used filtering technology to extract road points from elevation-based information with the extraction of curbstone points based on vertical points monitored by a Hough transform. These methods are more suitable for structural road types, results are poor for irregular roads with varying road widths. 

To obtain high-resolution road information for irregular structural roads, Yang et al. [2] and Fang and Yang [38] extracted three types of structural roads using extracted ground and curb points in three adjacent moving windows based on density, slope, and height jump. Each type of curb; however, had corresponding models and thresholds and this method is only applicable to structural roads with curbstones. Yadav et al. [39,40] filtered ground points based on height difference and detected road surface points based on surface roughness, topology, and the density of laser points for the specific structural road type. They then refined the final road edge based on a best-fit polynomial. Yuan et al. [41] used the maximum entropy of fuzzy clusters to extract the straight lines that belong to the road surface for each scan line based on the slope angle and location. Although this method works well regardless of road edge regularity or type (structural and semi-structural), it also considers location and slope angle. Yang et al. [42] proposed a 3-D local feature binary kernel descriptor method to extract road information based on the shape and intensity information of the mobile laser points. The binary kernel descriptor extracted road information by coding the shape and intensity of the 3-D laser points in a random forest classifier with a combination of binarization components and Gaussian kernel density estimations.

For other extraction methods based on intensity images, which are generated from laser data, Husain et al. [1] semi-autumnally extracted road boundary lines based on intensity images generated from laser points. Balado et al. [43] used planar segmentation based on a split and merge operation method with geometric and topological information. However, this method is affected by the quality of the input data and is unable to segment small elements. Zai et al. [44] extracted road edges based on the super voxels and graph cuts method, in which super voxels choose smooth points as seeds and assign points into facets centered on seeds based on geometric intensity and spatial distance attributes. The final road edge is extracted based on the α-shape and graph cuts with energy minimization. This method is supervised classification and requires specification of positive and negative backgrounds. Anttoni et al. [26] introduced an image-processing algorithm to extract road lines and markings from intensity images and curbstones from height images based on 3-D laser points. Although these methods yielded promising results, they were only performed on simple structural road types, such as flat roads with perpendicular curbstones. To extract non-structural road information, Kumar et al. [9] used a combination of gradient vector flow and active contour models with a balloon parametric function to extract road edges from a 2-D raster surface based on the hypothesis that attributes, such as reflectance, elevation, and pulse width, can distinguish road information from curbstones and grass–soil edges. However, extracted structures require further refinement. 

Based on the above description, most methods rely on conventional parameters (i.e., slope, point density, and road width), and few methods simultaneously focus on extracting structural and non-structural road edges. In this study, we develop an automatic method to efficiently and accurately extract structural and non-structural road edges from large-scale MLS data. For high-precision road extraction, we introduce a topological network of point clouds, which was built by adopting adjacent scan lines. This network was used to rapidly acquire road and curb points to extract high-resolution road edges. Compared with previous methods, the method proposed in this study can handle large-scale datasets to account for different structural and non-structural road types and complex road conditions (e.g., median inlands and pedestrians) without dependence on conventional parameters (i.e., slope, point density, and road width). Most thresholds in this method have identical stability and are not influenced by traditional parameters, such as slope, road width, density, and elevation.

## 3. Key Technology and Algorithm

**Figure 1:**
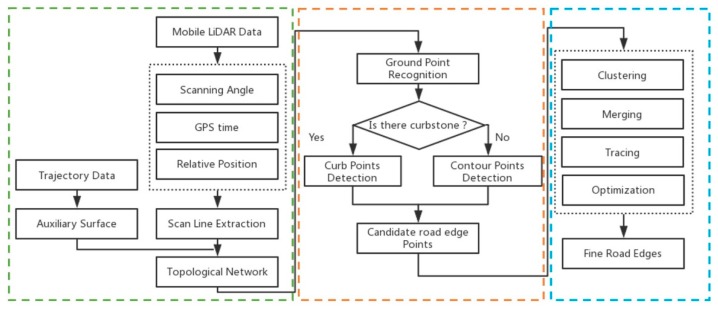
A framework for the extraction of road edges from the MLS data.

The framework for the proposed method is shown in Figure 1. The approach focuses on the extraction of fine road edges from MLS data and is divided into four steps. First, the topological network of laser points is constructed based on the scan line. Second, ground points are recognized based on the topological network. Third, curb points are detected based on the topological network and the auxiliary surface, after which candidate road edge points are identified. Finally, road edges and refined road points are determined. 

### 3.1. Topological Network Construction

The key point of the proposed method is the construction of a topological network based on MLS data. However, constructing a network based on unorganized 3D laser points is difficult and time-consuming [2]. Ibrahim and Lichti [25] used the K-D tree data structure method to organize laser points, and points selected as query points were used in the neighborhood search. In this study, the topological network was defined as a new spatial retrieval structure between adjacent laser points, constructed by using optimal neighbor points between adjacent scan lines. The purpose of constructing a topological network is not only to effectively manage and organize the irregular distribution of laser points, but also to extract road features used to simplify the extraction method, reduce extraction thresholds, and eliminate interference from traditional parameters. 

Accordingly, the extraction of scan lines [2,38,45] is the first step in constructing the network. Given that MLS is based primarily on the work mode of linear scanning, the same object will show similar spatial distribution characteristics in adjacent scan lines. Moreover, as almost all consecutive laser points have a similar time interval or scan angle difference, the angle or time difference [2] between consecutive points can be used to partition scan lines. In this study, the extraction of scan lines was based on scan angle differences between consecutive laser points, as shown in Equation (1). When the scan angle presents an interval jump [2], it can be determined that point $P_{i}$ is the termination point of the scan line and point $P_{i+1}$ is the starting point of the next scan line. Based on the scan angles of consecutive laser points, we found that the scan angle range of laser points is located at [$\theta_{min}$, $\theta_{max}$], where Δθ represents the threshold of the angular difference, that is, $\Delta \theta =360^{{}^{\circ}}-\theta_{max}+\theta_{min}$.

(1) $$\left|P_{i+1\left(angle\right)}-P_{i\left(angle\right)}\right|>\Delta \theta$$

If there is no scan angle for each laser point, the scan line could be extracted based on the GPS time for each laser point. Similarly, when the difference of GPS time between adjacent points ($P_{i}\;and\;P_{i+1}$) presents a time interval jump, it can be determined that point $P_{i}$ is the termination point of the scan line and point $P_{i+1}$ is the starting point of the next scan line. 

**Figure 2:**
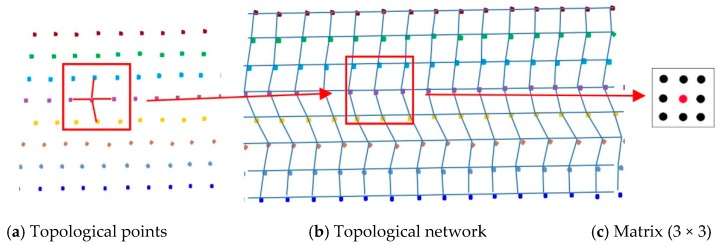
Construction of the topological network; different colors represent different scan lines.

Based on the extracted scan line, we defined the topological points as the optimal spatial neighbor points between adjacent scan lines and the previous and next points of the same scan line, as shown in Figure 2a. The topological network was constructed based on topological points (Figure 2b). Each point has a maximum of four optimal neighbor points and is limited to a one-to-one topology in a single direction. For each point, a matrix can be constructed based on its topological points, as shown in Figure 2c.

Given that each scan line has a small number of laser points, the optimal adjacent point for each point can be easily found among the adjacent scan lines. This condition simplifies and accelerates construction of the topological network. The subsequent extraction process can then be handled easily based on this network.

### 3.2. Recognition of Ground Points

**Figure 3:**
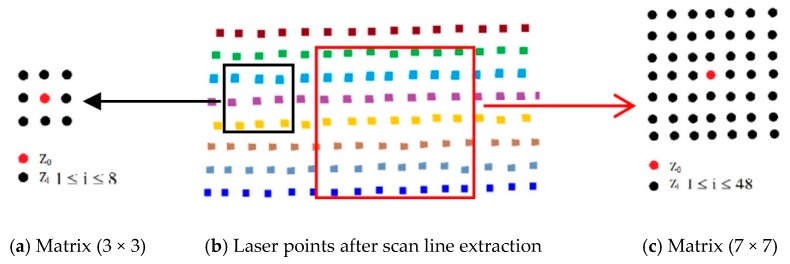
Definition of approximate matrices.

The complexity of road infrastructure and the surrounding road corridor environment—trees, buildings, vehicles, and pedestrians—increases the difficulty in extracting ground and curb points. In this study, ground points were extracted using a moving window combined with the topological network. The moving window comprises the current laser point and its topological points. We used the moving window by considering the current point and its topological points as an approximate matrix (Figure 3) to achieve the extraction of ground points based on Equations (2) and (3). Equation (2) was used to extract points located on the same planar (e.g., ground, pavement, flat roof, or top of a car) based on the sum of elevation differences between the center point and its topological points in the matrix. Equation (3) was used to extract points at a specified elevation. The specification of the matrix can be 3 × 3 (Figure 3a), 5 × 5, or 7 × 7 (Figure 3c). To accelerate calculations, a 3 × 3 matrix is usually selected. Ground points were subsequently calculated using Equations (2) and (3).

(2) $$\sum_{i=0}^{i<n^{2}-1}\left|Z_{i}-Z_{0}\right|\leq \Delta z_{1}$$

(3) $$\left|Z_{0}-z_{s}\right|<\Delta z_{2}$$

where Z is the laser point elevation, the subscript 0 denotes the center point of the matrix, the subscript i represents the topological point of the matrix center point, Δz1 is related to the density of laser points and road roughness, which has an initial value of 0.05 m related to the empirical value—if the ground point density is large, the threshold value is small, otherwise it is larger (both the ground roughness and the Δz1 increase), $z_{s}\;$ is the elevation of the road point under the scan car based on scan angle. n represents the size of the matrix, and Δz2 is used to identify an elevation range to restrict the distribution of ground points, which has an initial value of 0.2 m. This method is achieved based on Δz1 and Δz2 to extract ground points without using the traditional parameters of intensity, density, and slope. The extracted ground points comprised mainly road points, including pavement points on both sides of the road. Meanwhile, curb points were not included. At the same time, trees, buildings, vehicles, and pedestrians were; therefore, effectively removed. For structural roads with different types of curbstones, the next step was the detection of curb points. For non-structural roads without curbstones, we identified ground boundary points as the location of the road edge, so that contour points for the ground were considered candidate road edge points.

### 3.3. Detection of Curb Points 

**Figure 4:**
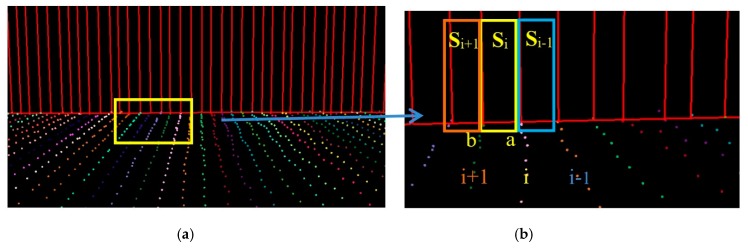
Trajectory auxiliary for each scan line (**a**) and definition of each auxiliary surface (**b**); a and b: Points in i and i+1 scan lines, respectively. Si: Auxiliary plane corresponding to i scan line.

For structural roads, given that the curb is located in the area between the road and green belts (or pavement), the curb was treated as the road boundary. Compared with a previous method [2], the extraction results of curb points were inevitably mixed with misclassified boundary points, such as wheel points, which have similar geometric characteristics to curbs. To avoid this phenomenon, we extracted curb points by using the moving window combined with the topological network based on non-ground points and auxiliary surface based on ground points. The moving window was constructed from laser and optimal spatial neighbor points, similar to the moving window described in Section 3.2. The construction of an auxiliary surface was based on trajectory data, that is, the vertically referenced surface. The auxiliary surface, as a reference surface, was used to extract curbstone points and was perpendicular to the scanning line. This auxiliary surface was divided into many adjacent planes by scan lines, as shown in Figure 4a. Each scan line corresponded to an auxiliary plane, as shown in Figure 4b. Points a and b represent the points in the i and i+1 scan lines, respectively. Plane Si is the auxiliary plane corresponding to the i scan line, expressed as $A_{i}X+B_{i}Y+D_{i}$ = 0, where variables $A_{i}$, $B_{i}$, and $D_{i}$ are parameters of the Si auxiliary plane.

Based on the auxiliary surface and topological points, curb points were extracted using Equations (4)–(6):

(4) $$\sum_{i=0}^{i<n^{2}-1}\left|d_{i}-d_{0}\right|\leq \Delta d$$

(5) $$\left|Z_{0}-z_{s}\right|<\Delta z_{3}$$

where

(6) $$d_{i}=\frac{\left|A_{i}\cdot X_{i}+B_{i}\cdot Y_{i}+D_{i}\right|}{\sqrt{A_{i}^{2}+B_{i}^{2}}}$$

where $d_{i}$ is the distance between the point of the moving window and its corresponding auxiliary plane; subscript 0 denotes the center point of the moving window; and subscript i is the point corresponding to the center of the square matrix. Equation 4 is similar to Equation (2), in that both identify laser points in a 3 × 3 matrix located on the approximate vertical plane. Equation (5) is used to control the extracted curb points within the specified elevation range. The value of Δd is directly proportional to the roughness and density of the extracted surface. The initial value of Δd is 0.1 m based on the empirical value. Although the threshold Δd is directly related to the surface roughness, the point density also affects the threshold to some extent. Under the same rough surface, the higher the point density is, the smaller the threshold value is. The Δz3 value is used to identify an elevation range with which we restrict the distribution of curb points. 

**Figure 5:**
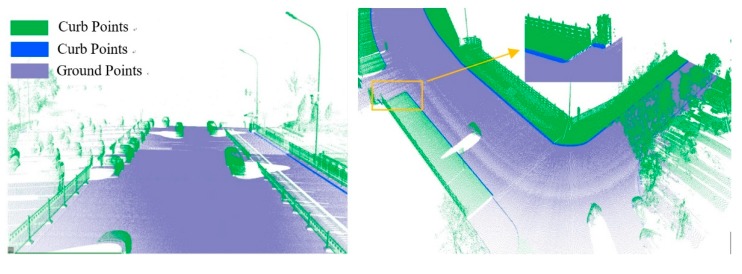
Extraction results for ground and curb points, curb points in blue, ground points in gray, and non-ground points in green.

As shown in Equation 6, the extraction of curb points in this step depends primarily on two thresholds (Δd and Δz3), without using traditional parameters such as angle, slope, and density. Based on the thresholds of Δd and Δz3, the misclassified boundary points (e.g., pedestrian and wheel bottom points) can be effectively eliminated, because the surface roughness of wheel and pedestrian surfaces are larger than the curb surface. Curb points extracted by the proposed method are illustrated in Figure 5. Based on the extraction result of road and curb points, we identified candidate points for the road edge, which are located at the intersection of the road and curb points.

### 3.4. Extraction of Road Edges

In this study, we extracted road edges based on the detection of candidate road edge points. For structural road types, we treated the intersections of road and curb points as road edge points, whereas for non-structural road types, we treated the contour points in the ground points as the location of the road edge. Non-edge points with similar geometric characteristics and similar surface roughness to those of curb points (e.g., fence bottom sides within the road area) increased the discontinuous phenomenon of road edge points. It; therefore, becomes necessary to extract road edges and refine the road area. Road edge extraction was accomplished through the following three steps:

(1) Clustering. The K-nearest neighbor method [2] was used to divide candidate edge points into clusters. At the same time, considering the similar geometrical properties between the adjacent parts of a road boundary, we used the Euclidean distance between edge points and the auxiliary surface as the distance metric to assist clustering. If points were located on the same road edge, the distance difference (Δdist_p) of adjacent edge points was less than 0.1 m.
(2) Merging. Clusters in the same road boundary were merged using two distance metrics: The distance difference (Δdist_c) between the cluster and auxiliary surface, and the minimum distance (Δdist_min) between two clusters. We used Δdist_c to identify whether clusters were located on the same road edge. If they were, and if Δdist_min was within a selected value, we merged the clusters.
(3) Tracing and optimization. Given the similar geometries for a given edge, we performed optimization using the auto-check, auto-repair, and auto-fill methods. Auto-check was used to identify and eliminate non-road edge points and clusters from candidate road edge points. We identified non-road edge points with large distance differences between the point and auxiliary surface compared with other candidate road edge points located in the same cluster and non-road edge clusters with fewer points if the number within the cluster was less than N. Here, N is the number threshold of laser points in a cluster.

**Figure 6:**
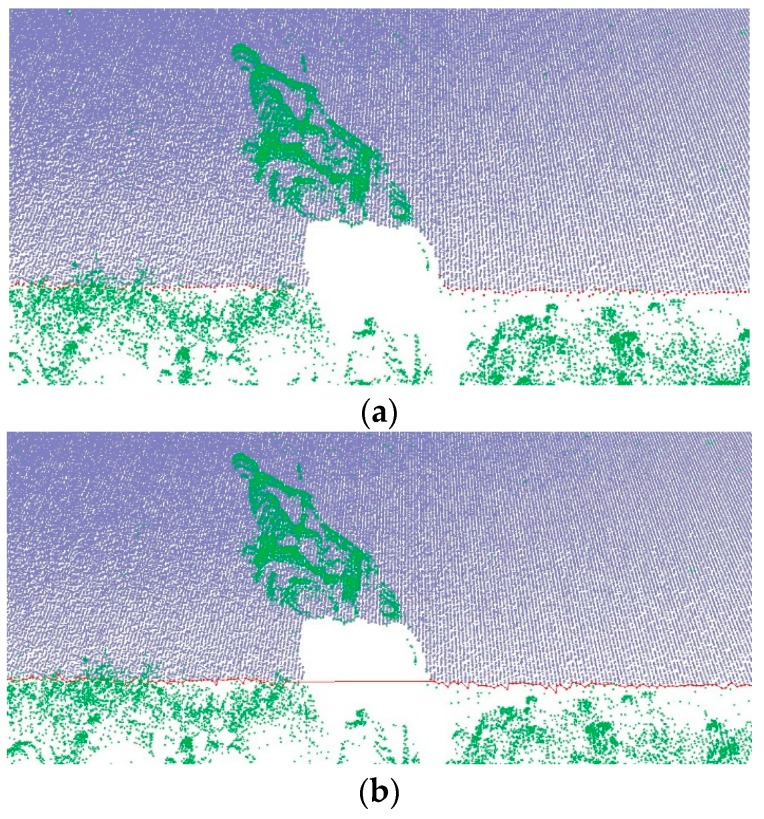
Extracted road edges of the grass–soil road; road edge points are plotted in red, and road edge is indicated with the red line. (**a**) Road edge points; (**b**) road edge.

**Figure 7:**
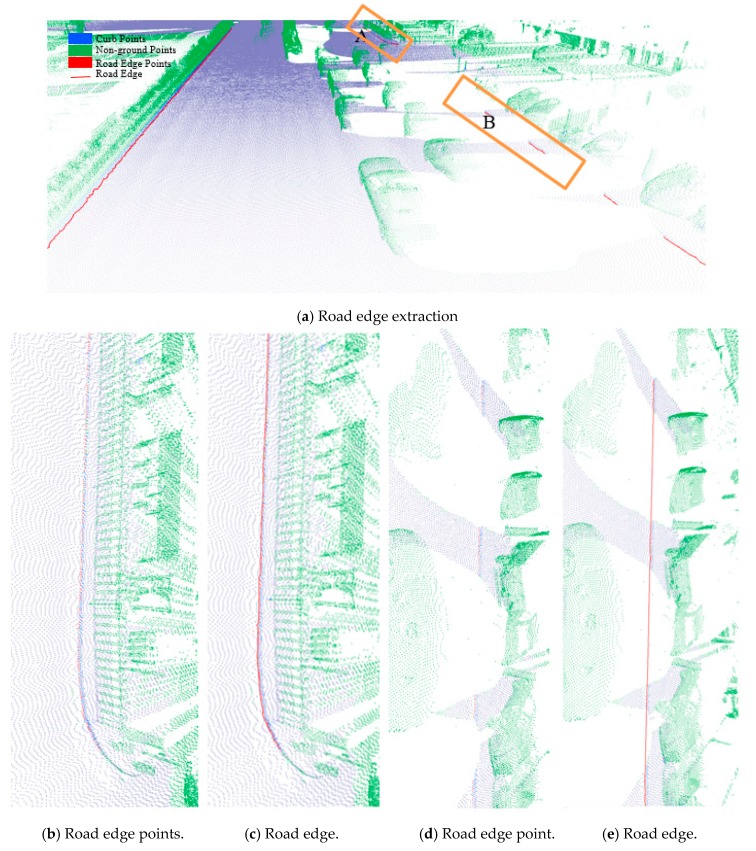
Extracted road edges for a structural road. (**b**) and (**d**) are the close-up view of region A and B in (**a**), respectively; and (**b**) and (**d**) show the road edge points extracted based on our proposed method; (**c**) and (**e**) are the road edge of (**b**) and (**c**), respectively.

Auto-repair was used to remove non-road edge points and clusters, connect these adjacent points within a certain distance on the same road side, and fit the point–point connect line. We used auto-fill to extract new road edge points with characteristics similar to those of the previously identified road edge line to fill small data gaps based on the interpolation method and generate a complete road edge line by judging the slope of both road edge sides of the hole. If the gap exceeds a certain distance based on the actual road scene, the reference value is 3 m, it is considered as a large gap, making it difficult to meet the requirements by relying on adjacent road edges. Due to the overall consistency of road edge line trends on both sides, large gaps, mainly located on the other side road edge, tend to fill the gaps based on the interpolation method. Finally, the longest line was selected as the final boundary line on the same side of the road to eliminate fences and other boundaries. The extraction results for a grass–soil road are shown in Figure 6. The extraction results for road edges along a structural road are illustrated in Figure 7. 

## 4. Experiments and Analysis

### 4.1. Experimental Dataset 

**Figure 8:**
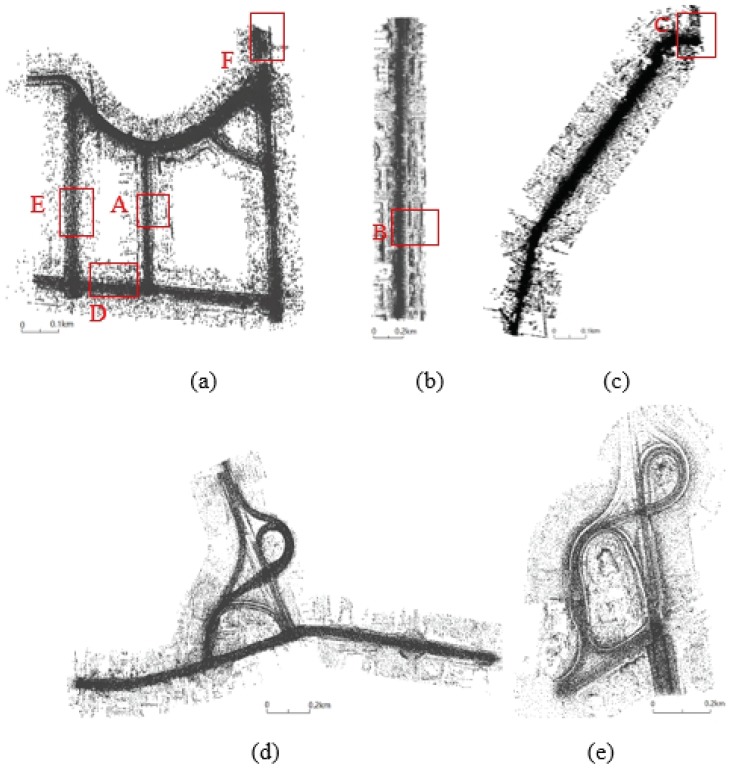
Experimental datasets of a suburban area from Tianjin (**a**), an urban area from Beijing (**b**), a rural area from Lianjiang (**c**), and structural (**d**) and non-structural (**e**) highway ramps in Taian.

Five representative experimental datasets of varying laser point densities and road conditions were selected to demonstrate the feasibility, practicality, and efficiency of the proposed method. These datasets were acquired using the SSW-IV [46,47] developed by the Chinese Academy of Surveying and Mapping. This system can be equipped with different types of laser scanners, such as the Chinese RTW or Riegl laser scanners. For the orientation of laser scanners in SSW-IV, the forward direction is defined as the X direction, vertical forward X direction as the Y direction, and vertical upward is the Z direction, it satisfies the right-hand criterion of coordinate system. For the Riegl laser scanner, we used the VUX-1HA mode to collect data with a scan speed of 250 scans/second, a survey-grade accuracy of 5 mm, and a measurement rate of up to 1,000,000 meas./sec with a ‘full circle’ 360° field-of-view that allows unrestricted data acquisition. For the RTW laser scanner, the scan speed is up to 100 scans/second, the survey-grade accuracy is less than 10 mm, the measurement rate is up to 500,000 meas./sec with a ‘full circle’ 360° field-of-view that allows unrestricted data acquisition. The experimental datasets include the following details:

(1) The dataset shown in Figure 8a originates from a complex suburban area located in Tianjin, China. We performed data acquisition using an SSW-IV with the domestic RTW laser scanner. The data consist of approximately 206 million laser points with a total length of 4.26 km. The mean laser point density is 694 p/m^2^. The scene has three types of curbstones (vertical, inclined, and arc curbs) as road edges. This dataset has a complex road environment with many occlusions and road entrances (occlusions and road entrances are challenges for road edge extraction).
(2) The dataset in Figure 8b shows an urban residential area in Beijing, China, characterized by a complex and occlusion-rich road environment that includes many cars, trees, fences, and pedestrians. The presence of these objects increases the difficulty of road extraction. This dataset was also acquired using the SSW-IV with the Riegl laser scanner. The mean laser point density is 7416 p/m^2^ and the number of laser points in the scene total to 164 million with a length of 1.9 km.
(3) Figure 8c shows a non-structural road dataset from a rural area of Lianjiang, China, which was acquired using the SSW-IV with a domestic RTW laser scanner. The mean laser point density is 1717 p/m^2^ and the number of laser points in the scene total 36 million with a length of 1.07 km.
(4) The datasets in Figure 8d,e show highway ramps for structural and non-structural road types in Taian, China, respectively, and were acquired using the SSW-IV with a Riegl laser scanner. The mean laser point density is 867 p/m^2^. Laser points in Figure 8d total 54 million with a length of 4.08 km and those in Figure 8e total 34 million with a length of 2.8 km.

### 4.2. Means of Validation 

Road edges extracted by the proposed method were compared with manually-digitized road edges by calculating their correctness (Equation (7)), completeness (Equation (8)), and quality (Equation (9)) [2,9].

(7) $$\mathrm{correctness}=\frac{\sum_{i=0}^{k-1}ET}{\sum_{i=0}^{k-1}ET+\sum_{i=0}^{p-1}EF}$$

(8) $$\mathrm{completeness}=\frac{\sum_{i=0}^{k-1}ET}{\sum_{i=0}^{k-1}ET+\sum_{i=0}^{m-1}EL}$$

(9) $$\mathrm{quality}=\frac{\sum_{i=0}^{k-1}ET}{\sum_{i=0}^{k-1}ET+\sum_{i=0}^{m-1}EL+\sum_{i=0}^{p-1}EF}$$

where ET is the length of the extracted road matching the reference road edges; EF is the length of the extracted false positive road; EL is the length of the non-extracted road; k is the number of the automated true road edges; p is the number of the extracted false positive roads; and m is the number of non-extracted roads. A buffer zone [9] was introduced to identify whether extracted and manually-digitized road edges were coincidental. The buffer zone used the manually-digitized road edge as the centerline to determine left and right buffer edges. If an extraction result was located at the buffer zone, the road edge was then regarded as true; if not, it was then false.

### 4.3. Results and Discussion

To validate the proposed method, extraction results for the road edge were quantified using the parameters shown in Table 1. During the optimum parameter selection process, three factors were considered: 1) Density; 2) the road structure type (i.e., the parameter for parameters with different road structures is different); and 3) the actual road scene, such as vehicle occlusion. The proposed method was calculated using a computer with 8 GB of RAM and an intel(R) Core (TM)i7-8550U CPU @1.80GHz running the VS2010 C/C++ language. 

**Figure 9:**
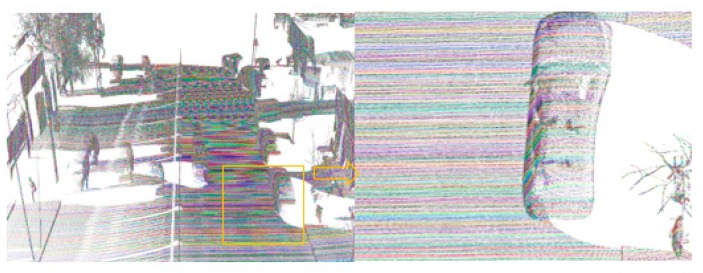
Extraction results for scan lines; different colors represent different scan lines.

Extraction of the scan line is based on differences in the angles of laser points. The scan angle range of each scan line for these five datasets was 60°–320°, the threshold of ∆θ is 100°. The extraction result of scan lines is shown in Figure 9. The topological network was constructed based on scan lines.

In the extraction process, the extraction of ground points involves two critical thresholds (Δz1 and Δz2) based on the moving window (3 × 3). Similarly, extraction of road curb points also has two critical thresholds (Δd and Δz3) based on the moving window. Emphasis is placed on the critical thresholds in the following discussion. The detailed relationships between the thresholds and the extraction results are shown in Table 2 and Table 3, using the arc curb identified by box A in Figure 8a as an example. To further investigate the relationship between thresholds (Δz1 and Δz2) and extracted ground results, a height amplification factor of 10 was used to amplify the details of the extraction results (Table 2). 

As shown in Table 2, when Δz2 remains constant, Δz1 continues to increase. This increases the density of the extracted ground points, and the range of ground points grows on both sides of the road. More specifically, when Δz2 is 0.2 and Δz1 is 0.01, the extraction result is a flat set of laser points, with uneven road surface points having been excluded. When Δz2 is 0.2 and Δz1 is 0.1, the extraction result contains uneven road surface points. These trends occur because the threshold of Δz1 is related to the roughness of the extracted surface, and Δz2 defines the maximum elevation plane to restrict the extraction result. The relationship between the extraction results and thresholds for curb points is shown in Table 3. 

**Figure 10:**
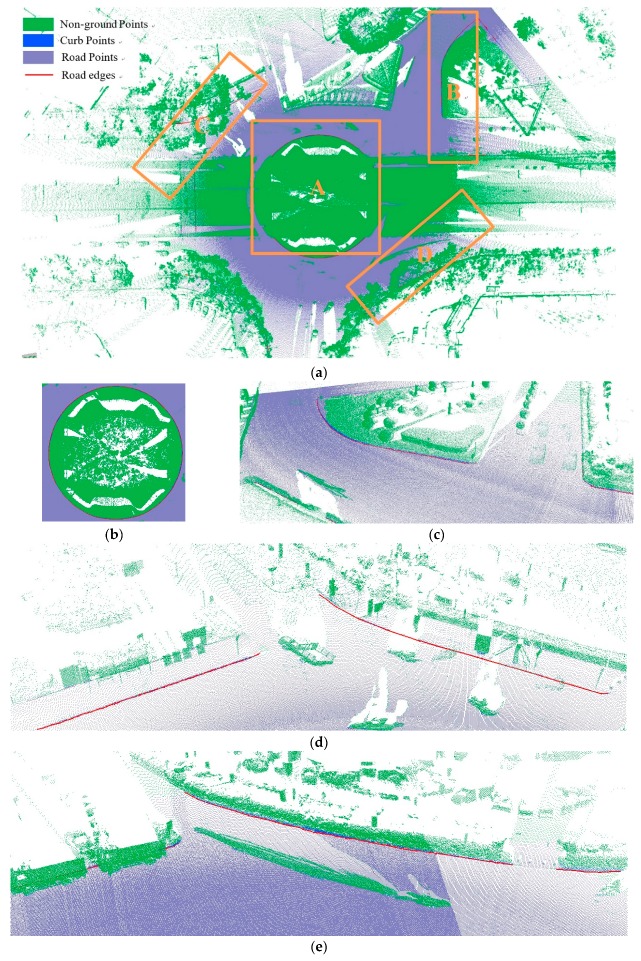
Extraction results based on the proposed method. (**a**) Extraction result; (**b**) Sample A; (**c**) Sample B; (**d**) Sample C; (**e**) Sample D.

The threshold of Δd has a function similar to the threshold Δz1, and Δz3 is similar to the ground extraction threshold Δz2. The parameter Δd is related to the roughness of the extracted curbstone surface, and Δz3 is used to define the elevation range to restrict the extraction of curb points. The extraction result of the curb points for the structural road type when Δd and Δz3 are 0.3 and 0.1, respectively, is shown in Figure 10, which is another dataset with many cars and curved curbs. More specifically, the extraction results of Samples A, B, C, and D in Figure 10a are shown in Figure 10b–e, respectively. According to Figure 10, there were five road entrances and occlusions (mainly due to moving cars), which is challenging for road edge extraction. Moreover, the road corners were the main components of the region. Based on our proposed method, we nonetheless obtained better experimental results.

To further verify the extraction results for structured roads, three types of road curbs and three regions within the experimental suburban area were selected. These areas, each of which has a unique type of road curve, are, respectively, denoted by boxes D, E, and F in Figure 8a. Extraction results for each of the three types of road curbstones are shown in Table 4 for each region. Results display ground and curb points, road edges, the overlap map, and the local effects. The data suggest that ground points, curb points, and road edges were well extracted.

**Figure 11:**
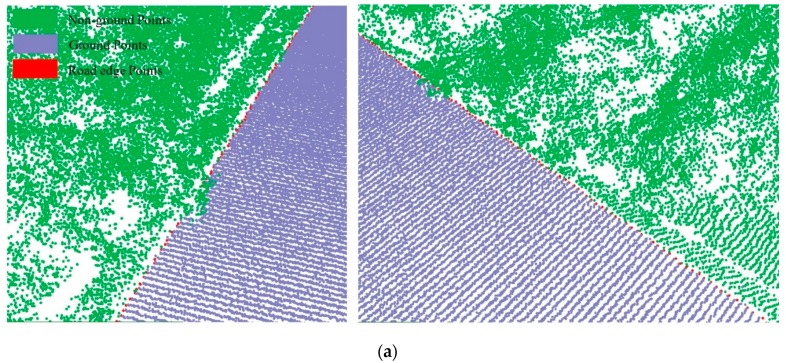

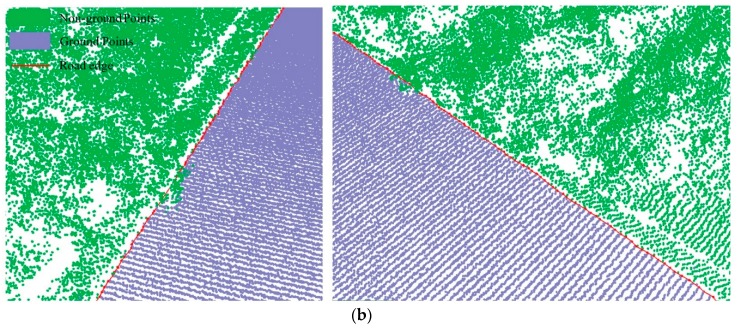
Extraction results for ground and curb points along a grass-soil road type. (**a**) Road edge points for grass-soil road; (**b**) Road edge for grass-soil road.

For the non-structural road, we successfully extracted ground points because the roughness of the road surface was significantly different to that of the roadsides. Extraction results for a non-structural curb are illustrated in Figure 11 and marked by box C in Figure 8c. The extraction results for the ground points are shown in Figure 11a, and the road edges are shown in Figure 11b. The results confirm that the method successfully extracted ground points and the road edge for a non-structural road type.

**Figure 12:**
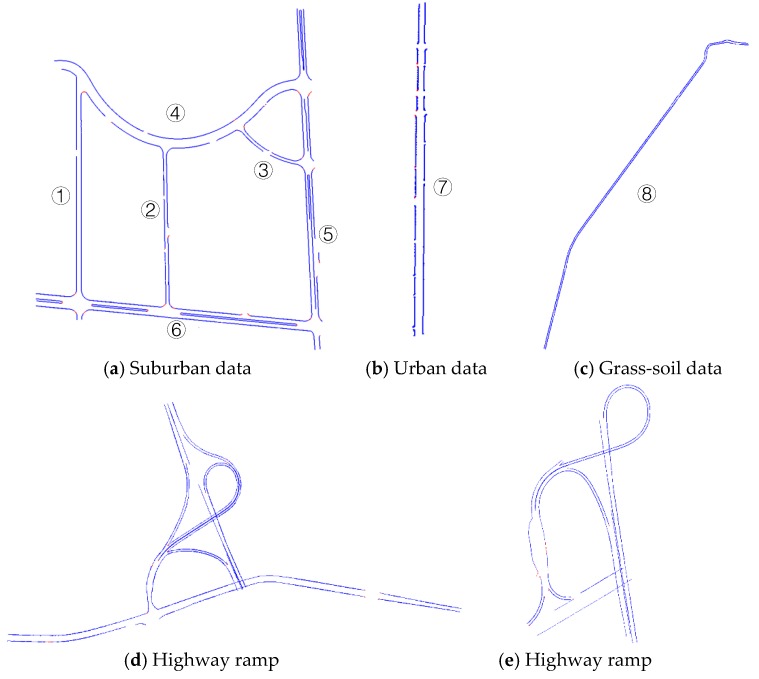
Extraction results for road edges; non-extracted road edges are indicated in red, and extracted road edges are indicated in blue.

The road edges extracted from these experimental datasets based on the proposed method are shown in Figure 12. Thresholds in the process of road edge extraction are shown in Table 1 for all experimental datasets. Figure 12a shows the extraction results for suburban roads using the proposed method. Non-extracted edges occur at locations with large turn angles. Figure 12b presents the extraction results from the urban dataset, and Figure 12c shows extraction results for the road edge in a rural non-structural grass–soil road. Figure 12d,e shows the extraction results for the road edge of the highway ramp.

The proposed road edge extraction method successfully extracted edges for both sides of the road for all datasets. The completeness, correctness, and quality of results were computed using Equations (7)–(9), respectively, to validate the road boundary extraction results. The assessment results are based on the buffer zone of the manually defined road edge (i.e., a 0.1 m buffer width). The detailed completeness, correctness, and quality results, together with related thresholds for the larger-scale experimental dataset (Figure 12), are listed in Table 5. Among the results, road 1–4, 7, and 8 show the results of each side of the road. Road 5 and 6 not only show the result of each edge, but also contain the edge result of the road median. Ramp (d) and (e) show the unified evaluation of the whole road.

Assessment results based on Table 5 show that the correctness of the extraction results for the structural suburban and urban road data using the proposed method were greater than 97.7%, completeness results were greater than 91.7%, and the quality measure values were greater than 90.9%. Correctness for the non-structural rural road was greater than 95.5%, completeness was greater than 96.2%, and the quality measure value was greater than 94.1%.

**Figure 13:**
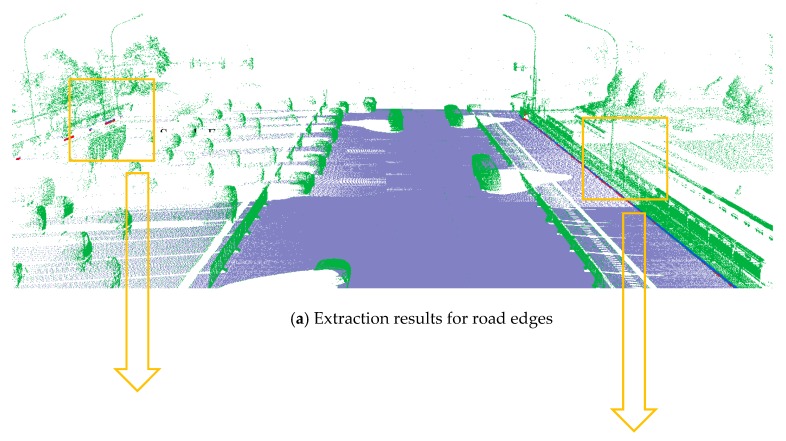

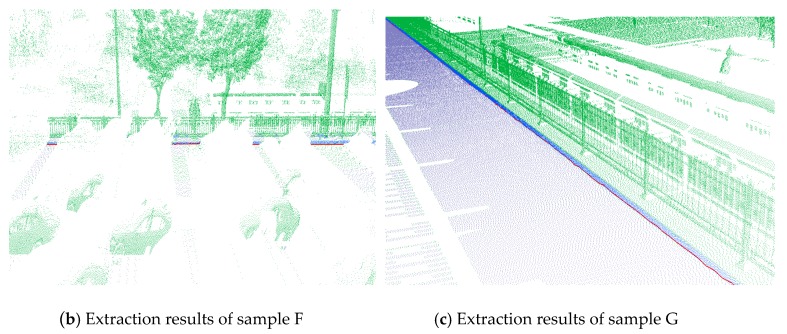
Extraction results.

A comparison of the assessment results with the previously developed methods [2] based on the validation method described previously (see Section 4.2) is listed in Table 6. For the structural road data (i.e., suburban and urban data), the correctness, completeness, and quality of the assessment results were higher than those obtained using the previous method [2], except for the completeness of suburban data. For the urban data, in particular, which had complex road conditions (i.e., many obstructions from cars, pedestrians, and fences), the assessment results based on our proposed method were much better than those obtained by the previous method [2]. Extracted road edges, denoted by box B in Figure 8b, are illustrated in Figure 13. Road points acquired within the road area are based on the extracted road edges. As shown in Figure 13b, road edges were still successfully extracted by the proposed method despite the influence of cars, pedestrians, or other objects. The entire road edge was easily acquired based on similar geometric road boundaries. For non-structural roads without a curb (i.e., rural data), our proposed method also achieved high assessment results for correctness, completeness, and quality values of 96.3%, 99.9%, and 95.9%, respectively. However, the previous method [2] is not suitable for road edge extraction on non-structural roads because it is based on the thresholds of height jump, slope, and density to identify road curbstones. In contrast, our proposed method is mainly based on the roughness of the extracted surface. 

For our proposed method, correctness values were high because road edges were acquired from curb points located at intersections with ground points for the structural data and from the ground boundary points for non-structural data. The calculation of completeness and quality was related to the non-extracted section, which was primarily concentrated on the larger part of the curved road, such as at road turns and residential entrances. This can be attributed to the fact that the extraction of curb points was not sensitive to road curve. As mentioned in Section 3.3, the road curve affected the extraction result. However, for Figure 10, which shows many road curves, road entrances, and occlusions, the extraction result is better. This is because the region depicted in Figure 10 has a high density. Subsequently, for the road turns, we could achieve high-resolution extraction results. Hence, the density and road curves were closely related to the extraction results. The curbstone points cannot be successfully extracted from road curves with lower density, which leads to failure to extract road edges. If the density increases, the accuracy of extraction results in corners will increase accordingly. 

There are two main reasons for road corner extraction failure. The first is the influence of density (i.e., the higher the density, the more the extraction completeness increases). The second is the occurrence of data holes caused by vehicle occlusion during data collection. Point cloud density is influenced by several parameters, such as point frequency, line frequency, and speed, among which the point and line frequencies are influenced by the specific type of equipment, and the speed is a subjective factor that has great influence on the point density during the data acquisition process. If the vehicle slows down, the laser point density increases and the point density decreases. Therefore, to improve the accuracy of corner extraction, the following two points in the data acquisition process require strict accordance with the precision requirements of the actual engineering: 1) Peak periods during mornings and evenings should be avoided, which ensure the absence of occlusion via vehicles and pedestrians; and 2) for road curves, the speed should be appropriately reduced to ensure a relatively high point density at corners, which help enhance the completeness of the road boundary extraction. 

In summary, the proposed method has several advantages. First, this method can be used not only in the accurate extraction of large-scale structural road edges, but also large-scale non-structural roads. Second, the method is implemented based on surface roughness without directly computing attributes such as slope, angle, and density. Moreover, it is effective regardless of whether the road width is fixed, the road is regular, or pedestrians and vehicles are present. Fourth, the correctness, completeness, and quality values associated with the extraction results are high and exceed those of previous methods. The assessment results are highly consistent across varying road conditions (i.e., complex road conditions with cars, trees, fences, pedestrians, and different curb types), which demonstrates the stability of our method. However, this method is not sensitive to road bending degree, leading to poor road edge assessment results when the road has a relatively sharp bend. At the same time, based on the extraction results of road edges, the road centerline, road width, cross slope, and longitudinal slope can easily be achieved.

## 5. Discussion

This study proposed a robust method to automatically extract structural and non-structural road edges from MLS data. The road edges, as well as road and curb points, were successfully extracted based on the construction of a topological network of laser points and auxiliary surfaces without using height jump, density, or slope. The successful extraction of road edges from large-scale experimental datasets verifies the feasibility, stability, and practicality of the proposed method. Quantitative analyses of the results obtained from these structural and non-structural datasets indicate that the correctness, completeness, and quality measure values exceed 95.5%, 91.7%, and 90.9%, respectively. 

This method can be used not only to extract large-scale structural road data with severe pedestrian or vehicle occlusion, but also to extract large-scale road edges for non-structured roads (e.g., grass–soil road types). At present, our proposed method is being ported and tested on a private cloud platform to accelerate computing for improved efficiency. Our future research will focus on how to enhance the overall applicability of the proposed approach, particularly for special road conditions with large bending degrees. Additionally, these results are based on measuring various geometries with one type of laser scanner; future work would benefit from testing other sensors. 

## Figures and Tables

**Table 1:** Parameters used for calculation by the proposed method.

Parameter	Value				
	Suburban Data	Urban Data	Rural Area	Ramp (d)	Ramp (e)
∆*θ* (°)	100	100	100	100	100
Δ*z*_1_ (m)	0.05	0.05	0.05	0.05	0.05
Δ*z*_2_ (m)	0.2	0.2	0.2	1.0	1.0
Δ*d* (m)	0.25 (Vertical curb)	0.1			
	0.15 (Inclined curb)				
	0.3 (Arc curb)				
Δ*z*_3_(m)	0.1	0.1			
Δdist_p (m)	0.1	0.1	0.1	0.1	0.1
Δdist_c (m)	0.3	0.3	0.3	0.3	0.3
Δdist_min	2	2	2	2	2
N	10	20	15	15	15
time(min)	25.12	19.87	4.52	6.81	3.67

**Table 2:** Relationships between the extraction result and thresholds for ground points.

Δz1/Δz2	Extraction Results	Amplified Height (×10)
0.01/0.2	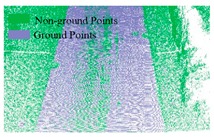	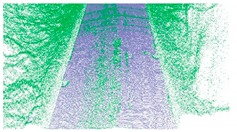
0.05/0.2	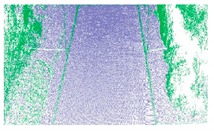	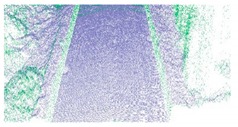
0.1/0.2	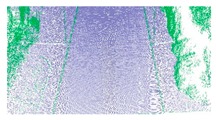	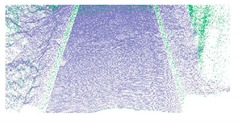
0.05/0.1	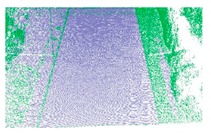	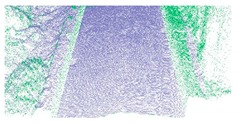
0.05/0.3	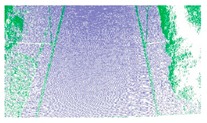	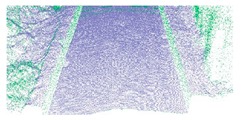

**Table 3:** Relationship between the extraction result and thresholds for curb points.

Δ*d*/Δ*z*_3_	Extraction Results	Curb Points Only
0.25/0.2	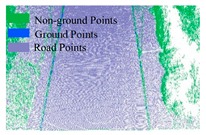	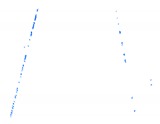
0.30/0.2	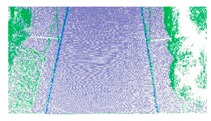	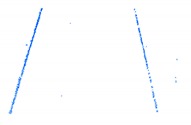
0.35/0.2	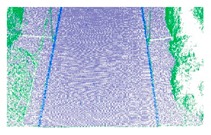	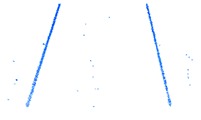
0.30/0.1	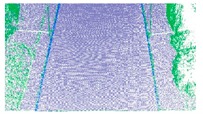	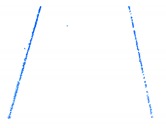
0.30/0.3	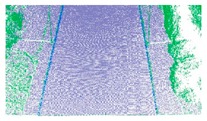	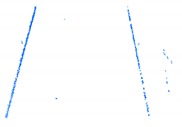

**Table 4:** Extraction results for three different types of road curbs.

Position	D	E	F
Curb type	**Inclined Curb**	**Vertical Curb**	**Arc Curb**
	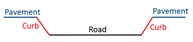	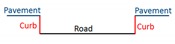	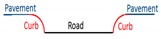
Ground and curb points	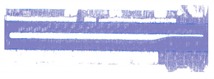	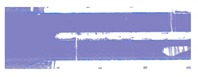	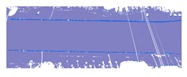
Road edge	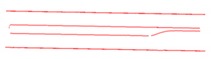	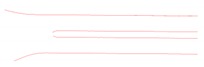	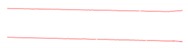
Overlay map	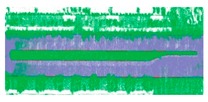	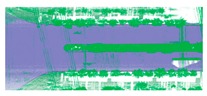	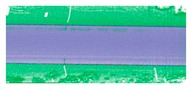
Local effect	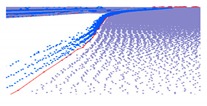	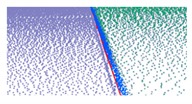	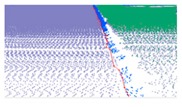

**Table 5:** Assessment results for suburban, urban, and rural areas.

Data		Road	Correctness	Completeness	Quality
Suburban	①	Left	0.993	0.967	0.960
		Right	0.993	0.961	0.955
	②	Left	0.977	0.941	0.920
		Right	0.994	0.955	0.949
	③	Up	0.983	0.924	0.909
		Down	0.995	0.926	0.921
	④	Up	0.991	0.959	0.951
		Down	0.990	0.947	0.938
	⑤	Left	0.984	0.941	0.927
		Middle	0.986	0.943	0.931
		Right	0.980	0.937	0.919
	⑥	Up	0.984	0.917	0.904
		Middle	0.985	0.930	0.916
		Down	0.979	0.974	0.955
Urban	⑦	Left	1	0.965	0.965
		Right	0.978	0.982	0.961
Rural	⑧	Left	0.971	1	0.965
		Right	0.955	0.998	0.953
Ramp (d)			0.978	0.962	0.941
Ramp (e)			0.981	0.977	0.959

**Table 6:** Assessment results compared with those from the previous method [2].

Dataset	Method	Assessment		
		Correctness	Completeness	Quality
Suburban	Proposed method	0.987	0.944	0.933
	Yang et al. [2]	0.981	0.946	0.929
Urban	Proposed method	0.989	0.974	0.963
	Yang et al. [2]	0.969	0.941	0.922
Rural	Proposed method	0.963	0.999	0.959
	Yang et al. [2]	---	---	---
Ramp (d)	Proposed method	0.978	0.962	0.941
	Yang et al. [2]	---	---	---
Ramp (e)	Proposed method	0.981	0.977	0.959
	Yang et al. [2]	---	---	---
